# Psychological capital, mindfulness, and teacher burnout: insights from Chinese EFL educators through structural equation modeling

**DOI:** 10.3389/fpsyg.2024.1351912

**Published:** 2024-03-14

**Authors:** Dongxia Liu, Ruikang Du

**Affiliations:** ^1^College of Foreign Languages and Literature, Jilin Normal University, Siping, China; ^2^Academic Affairs Office, Jilin Normal University, Siping, China

**Keywords:** English as a Foreign Language teachers, mindfulness, psychological capital, SEM, teacher burnout

## Abstract

**Objective:**

This cross-sectional study employs Structural Equation Modeling (SEM) to examine the relationships among psychological capital, mindfulness, and teacher burnout in a sample of 387 Chinese English as a Foreign Language (EFL) educators.

**Methods:**

Self-reported data were analyzed to investigate the direct and indirect effects of psychological capital on teacher burnout, with mindfulness serving as a potential mediator.

**Results:**

Our SEM analysis reveals a significant direct negative association between psychological capital and teacher burnout. Moreover, mindfulness significantly mediates the relationship between psychological capital and burnout, indicating that higher psychological capital leads to increased mindfulness, which subsequently reduces burnout.

**Conclusion:**

This study underscores the importance of psychological capital and mindfulness in mitigating teacher burnout among Chinese EFL educators. The findings suggest that interventions targeting both psychological capital and mindfulness practices could bolster teacher well-being and foster a more positive educational environment.

## Introduction

The teaching profession, while esteemed, faces unprecedented challenges in the modern educational landscape (Friedman, 1991), contributing to distressing levels of teacher burnout. This phenomenon, characterized by emotional exhaustion, depersonalization, and reduced personal accomplishment, not only imperils educators’ well-being but also detrimentally affects the quality of education and student outcomes (Maslach et al., 2001; Skaalvik and Skaalvik, 2010; Brunsting et al., 2014; Tsang et al., 2022).

Amidst mounting concerns about teacher well-being, there is an increasing need to delve into the factors that bolster resilience and mitigate burnout. This research investigates two crucial yet underexplored constructs: psychological capital and teacher mindfulness, aiming to shed light on their intricate interplay and impact within the realm of educator well-being. Psychological capital, often referred to as PsyCap, constitutes a reservoir of positive psychological resources—self-efficacy, hope, optimism, and resilience—that empower individuals to navigate challenges and adversities (Luthans et al., 2007; Gong et al., 2019). Within the context of teaching, higher levels of psychological capital equip educators with enhanced confidence in managing classroom complexities, maintaining optimism despite obstacles, nurturing hope in their students, and resiliently adapting to the demands of the profession (Zhang et al., 2019). Conversely, mindfulness, a distinctive yet complementary construct, involves cultivating present-moment awareness, non-judgmental acceptance, and emotional attentiveness (Kabat-Zinn, 1994; Capurso et al., 2014). Teachers practicing mindfulness develop heightened sensitivity to their mental and emotional states, enabling better stress management, emotion regulation, and a sustained sense of purpose amidst the challenges inherent in teaching (Roeser et al., 2013).

The formulation of the research questions and the identification of the research gap in this study were carefully constructed to address critical voids in the existing literature concerning the well-being of Chinese EFL educators. The formulation of these questions stems from a synthesis of prior research and observations of the teaching landscape, both globally and within the context of China. Firstly, the choice to investigate the nexus between psychological capital and teacher burnout was prompted by a recognized need to delve deeper into the psychological underpinnings that contribute to the well-being of educators. While existing literature acknowledges the importance of psychological capital, a nuanced understanding of its direct impact on teacher burnout, particularly within the Chinese EFL context, remains limited. This research seeks to bridge this gap by exploring the specific dimensions of psychological capital that play a pivotal role in mitigating burnout among Chinese EFL educators.

Secondly, the deliberate focus on the mediating role of teacher mindfulness was informed by the acknowledgment that mindfulness practices can serve as a potential mechanism through which psychological capital influences teacher well-being. Although mindfulness has gained attention in the broader literature on well-being, its specific interplay with psychological capital and its impact on teacher burnout, especially in the Chinese EFL teaching domain, has not been extensively studied. This study seeks to unravel this intricate relationship and contribute valuable insights into how mindfulness acts as a protective buffer against burnout in the presence of heightened psychological capital.

The research questions were, therefore, carefully designed to address these gaps and to provide a holistic understanding of the dynamics between psychological capital, mindfulness, and burnout among Chinese EFL educators. The study aims to go beyond the existing theoretical frameworks by empirically examining these relationships, thereby contributing novel findings to the field of educator well-being. Against this backdrop, the following research questions guided this study:

*RQ1*: Is there a statistically significant negative correlation between psychological capital and teacher burnout among Chinese EFL educators?

*RQ2*: Does teacher mindfulness mediate the relationship between psychological capital and teacher burnout among Chinese EFL educators?

## Literature review

### Teacher burnout

Teacher burnout, a pervasive concern in education, is intricately characterized by emotional exhaustion, depersonalization, and diminished personal accomplishment (Maslach et al., 2001; Chang, 2009). This multifaceted syndrome arises from chronic workplace stressors prevalent in teaching, encompassing high workloads, emotional demands, and challenging student behaviors (Maslach and Leiter, 2016; Li, 2023). While the existing literature provides valuable insights into the dimensions, origins, and impact of teacher burnout (Shen et al., 2015), a critical examination reveals the need for a more nuanced understanding and targeted interventions within the context of Chinese EFL educators.

The dimensions of teacher burnout – emotional exhaustion, depersonalization, and diminished personal accomplishment – delineate its nuanced nature within the professional context (Maslach and Leiter, 1999; Maslach et al., 2001; Hastings and Bham, 2003; Maslach, 2003). Emotional exhaustion captures chronic feelings of emotional depletion and fatigue, highlighting the struggle to meet the emotional demands of teaching. Depersonalization manifests as a detached attitude toward students and colleagues, potentially diminishing the recognition of students’ distinct needs and emotions. Diminished personal accomplishment reflects a reduced sense of efficacy and achievement in the teaching role, posing a risk to professional self-worth (Madigan et al., 2023).

The origins of teacher burnout, traced back to the late 1970s through the work of Maslach and colleagues, emphasize the unique demands of the profession (Maslach, 1976; Maslach et al., 2001). However, a critical examination of these origins reveals the evolving landscape of education, with factors such as advancements in technology, changing societal expectations, and diverse student demographics shaping contemporary challenges for educators. The existing literature primarily rooted in the late 20th century may not comprehensively address the current stressors faced by Chinese EFL educators.

The impact of teacher burnout on educators’ well-being and the educational environment is undeniable (Kyriacou, 2001; Schaufeli and Bakker, 2004; Bakker et al., 2006; Zhang et al., 2023). Emotional exhaustion correlates with increased stress, anxiety, and depressive symptoms, while depersonalization hampers crucial teacher-student relationships. Diminished personal accomplishment diminishes job satisfaction and elevates turnover intentions, collectively eroding psychological well-being (Pyhältö et al., 2021). The consequences extend to the instructional quality and student experiences, with burned-out educators employing less effective teaching strategies, resulting in reduced student learning outcomes (Oliveira et al., 2021; Bing et al., 2022).

Despite the extensive literature on teacher burnout, a critical gap exists in understanding its specific nuances within the Chinese EFL educational context. Factors such as cultural influences, language barriers, and unique challenges faced by Chinese EFL educators remain underexplored (Cheng et al., 2023). This study aims to bridge this gap by exploring the relationship between teacher burnout, psychological capital, and mindfulness, offering culturally sensitive insights and effective strategies to promote the well-being of Chinese EFL educators. In doing so, it seeks to contribute to the broader discourse on teacher burnout and its implications for educational practices within the specific context of Chinese EFL education.

Furthermore, it is essential to recognize that the challenges faced by Chinese EFL educators extend beyond the traditional stressors outlined in the existing literature. Cultural nuances, language barriers, and the distinctive dynamics of language education add layers of complexity to the experiences of these educators. The current body of research often overlooks these unique factors, limiting the applicability of existing interventions and understanding of teacher burnout in this specific context. Exploring the interplay between psychological capital, mindfulness, and burnout within Chinese EFL education will not only enrich the literature on teacher well-being but will also provide tailored insights to inform interventions that resonate with the intricacies of this educational setting. As such, this study not only addresses the existing gap in literature but also aims to present a more comprehensive understanding of teacher burnout within the specific framework of Chinese EFL education.

### Psychological capital

In the quest to comprehend the determinants of teacher well-being, the construct of psychological capital, often termed as PsyCap, has emerged as a subject of considerable interest and scrutiny (Cheung et al., 2011). This multifaceted construct encapsulates several positive psychological attributes, prominently comprising self-efficacy, hope, optimism, and resilience (Luthans et al., 2007). Each component contributes distinctively to an individual’s psychological health and resilience when confronting adversity within their professional and personal spheres (Wu and Kang, 2023).

Self-efficacy, a fundamental component of psychological capital, represents an individual’s belief in their capacity to effectively accomplish tasks or attain specific objectives (Bandura, 1977). Rooted in social cognitive theory, self-efficacy stands as a foundational determinant of human motivation and performance, significantly impacting teachers’ perceptions of their abilities and influencing their pedagogical practices (Tschannen-Moran and Hoy, 2001). High levels of self-efficacy among educators engender a propensity to set ambitious goals, profoundly influencing student learning outcomes, and serving as a protective factor against burnout by empowering proactive approaches in navigating challenges (Skaalvik and Skaalvik, 2007; Xiyun et al., 2022).

Hope, another pivotal aspect of psychological capital, encompasses an individual’s cognitive orientation toward agency and pathways when striving for desired objectives (Snyder, 2000; Uwakwe et al., 2023). Within the teaching context, hope assumes a pivotal role in shaping educators’ commitment to their students and the educational process. Educators characterized by higher levels of hope tend to set ambitious objectives for their students and persist in their efforts to help them achieve these goals, contributing significantly to teachers’ resilience and psychological health, thus serving as a protective mechanism against burnout (Gilman, 2001; Feldman and Kubota, 2015; Lupșa et al., 2020).

Optimism, a critical facet of psychological capital, denotes an individual’s positive worldview, characterized by an anticipation of favorable outcomes and the belief that adverse events are transient and controllable (Scheier and Carver, 1992). Within the teaching profession, an optimistic outlook influences how educators respond to the challenges inherent in their roles. Teachers displaying higher levels of optimism tend to perceive difficulties as opportunities for growth and exhibit proactive problem-solving approaches, fostering resilience and serving as a preventive mechanism against burnout (Gilman, 2001; Vizoso et al., 2019).

Resilience, the final pillar of psychological capital, signifies an individual’s capacity to rebound from adversity, adapt to challenges, and maintain well-being despite setbacks (Luthans, 2002). This psychological resource is indispensable within the teaching profession, empowering educators to cope with the myriad stressors accompanying their roles. Resilient teachers exhibit enhanced emotional regulation, adaptability, and steadfast commitment to student success, contributing significantly to mitigating burnout (Riolli and Savicki, 2003; Gu and Day, 2007; Darvishmotevali and Ali, 2020).

The significance of psychological capital in promoting teacher well-being transcends the challenges encountered in the teaching domain (Barratt and Duran, 2021). Teachers confront diverse stressors, including heavy workloads and student behavioral issues, that can adversely affect their psychological health and precipitate burnout (Maslach et al., 1997; Kyriacou, 2001; Fathi et al., 2023a).

Psychological capital equips teachers with the necessary psychological resources to effectively manage these stressors. Self-efficacy instills confidence in navigating challenging classroom scenarios, while hope fosters direction and perseverance in the pursuit of educational objectives. Optimism encourages a positive outlook, facilitating proactive problem-solving, and resilience ensures the capacity to recover from adversities, collectively acting as preventive measures against burnout (Avey et al., 2011). These positive psychological resources, acting as protective factors, bolster teacher resilience, enhance emotional regulation, and thwart the development of negative emotional states linked with burnout. By empowering teachers with the requisite tools and mindset to confront challenges while preserving their psychological well-being, psychological capital contributes significantly to a more resilient, engaged, and contented teaching workforce. Consequently, the cultivation and nourishment of psychological capital assume paramount importance in promoting teacher well-being and preventing burnout.

Although existing studies have significantly contributed to unraveling the intricate relationship between psychological capital and teacher burnout, a critical analysis reveals specific gaps and nuances that necessitate further exploration. Zhang et al. (2019) elucidated the mediating role of coping styles in the influence of psychological capital and occupational stress on teacher burnout, providing valuable insights into coping mechanisms. Ferradás et al. (2019) explored diverse teacher profiles characterized by psychological capital, shedding light on the variability in burnout experiences among educators. Despite these valuable contributions, there remains a need to delve deeper into the contextual factors and individual differences that may modulate the impact of psychological capital on burnout within the distinct setting of Chinese EFL education.

Moreover, while Freire et al. (2020) emphasized flourishing as an intermediary between psychological capital and burnout, the broader cultural and contextual implications of well-being in the Chinese EFL teaching context require explicit exploration. Demir (2018) extended the understanding of psychological capital’s relationship with teacher well-being, yet a comprehensive examination of its application in the Chinese EFL educational landscape is conspicuously absent. Therefore, this study aims to bridge these gaps by focusing specifically on Chinese EFL educators, considering cultural nuances, language barriers, and unique challenges that may influence the interplay between psychological capital and burnout.

Kaya and Altınkurt (2018) provided valuable insights into the interplay of psychological and structural empowerment in connecting psychological capital and burnout, underlining the significance of empowering work environments. However, these insights may not entirely capture the specific organizational and cultural contexts shaping the experiences of Chinese EFL educators. This study seeks to expand on this by exploring how the organizational dynamics within Chinese educational institutions may modulate the relationship between psychological capital and burnout among EFL teachers.

Cheung et al. (2011) investigated psychological capital’s role as a moderator in the complex interplay of emotional labor, burnout, and job satisfaction among school teachers. However, the unique challenges faced by Chinese EFL educators, such as navigating a different linguistic and cultural landscape, may introduce distinctive emotional labor dynamics that warrant a focused examination. Therefore, this study aims to contribute to the existing literature by scrutinizing the moderating role of psychological capital within the specific context of Chinese EFL education.

In conclusion, while the aforementioned studies underscore the critical relevance of psychological capital in the broader understanding of teacher burnout, a careful examination reveals specific gaps and contextual variations that necessitate targeted exploration. This study aims to fill these gaps by providing culturally sensitive insights into the role of psychological capital in the burnout experiences of Chinese EFL educators, thereby contributing to a more comprehensive understanding of teacher well-being within this specific educational context.

### Mindfulness

Mindfulness, as a psychological construct, has gained remarkable recognition in recent years for its association with improved well-being, stress reduction, and emotional regulation (Kabat-Zinn, 1994, 2018, 2019). Originating from Buddhist traditions, mindfulness represents a state of being characterized by present-moment awareness and non-judgmental observation of thoughts and emotions (Kabat-Zinn, 1994). Adapted for contemporary contexts, including education, mindfulness emphasizes acknowledging experiences without judgment, fostering emotional regulation, and enhancing overall well-being (Grossman et al., 2004).

The evolution of mindfulness as a psychological construct traces its roots to mindfulness-based interventions (MBIs) developed from Eastern philosophies. Influential programs like mindfulness-based stress reduction (MBSR) and mindfulness-based cognitive therapy (MBCT) pioneered by Jon Kabat-Zinn and others played pivotal roles in popularizing mindfulness in Western psychology (Kabat-Zinn, 1982; Segal et al., 2002). Their incorporation of mindfulness meditation and cognitive therapy elements has demonstrated efficacy in various psychological domains, including stress reduction and depression prevention (Fathi et al., 2023b).

Within education, mindfulness practices have been adapted for teachers through programs like Cultivating Awareness and Resilience in Education (CARE) to enhance teacher well-being and address classroom challenges (Jennings and Greenberg, 2009; Xu et al., 2022). The utilization of mindfulness aims to equip educators with tools to manage stress and navigate professional demands effectively (Ergas and Hadar, 2023; Xie and Guo, 2023). The significance of mindfulness in teacher well-being is multifaceted and encompasses its potential to enhance emotional regulation, reduce stress, and mitigate teacher burnout (Hue and Lau, 2015). Mindfulness practices provide teachers with mechanisms to manage emotional and psychological demands more effectively, ultimately reducing emotional exhaustion and depersonalization—core components of burnout (Maslach et al., 2001; Mettler et al., 2023).

Mindfulness practices are notably influential in enhancing emotional regulation by encouraging non-reactive observation of emotional experiences (Chambers et al., 2009; Dariotis et al., 2023). This cultivation of awareness enables individuals to approach challenges with equanimity, respond with greater emotional intelligence, and navigate professional stressors more effectively (Roeser et al., 2013; McCaw, 2020; Mohammad Hosseini et al., 2023). Moreover, stress reduction through mindfulness—marked by reduced cortisol levels and enhanced relaxation—plays a pivotal role in preventing burnout, as stress is a primary precursor to teacher burnout (Skaalvik and Skaalvik, 2007).

Research consistently supports the role of mindfulness in preventing teacher burnout. Studies have shown associations between mindfulness and reduced emotional exhaustion, enhanced personal accomplishment, and lower depersonalization among educators (Jennings and Greenberg, 2009; Abenavoli et al., 2013; Sun et al., 2019). Moreover, mindfulness-based interventions have demonstrated the potential to reduce burnout symptoms and enhance teacher well-being (Jennings and Greenberg, 2009; Hwang et al., 2017). Additionally, mindfulness practices hold significance for classroom management and student outcomes. Teachers practicing mindfulness tend to create more positive classroom environments, fostering improved student-teacher relationships and positively impacting student learning outcomes (Jennings and Greenberg, 2009; Roorda et al., 2011).

While existing research has undeniably contributed to our understanding of mindfulness and its positive impact on teacher well-being and burnout, a critical analysis reveals specific gaps and areas for further exploration. Flook et al. (2013) demonstrated the efficacy of mindfulness interventions in alleviating stress and burnout symptoms while enhancing teaching efficacy, providing valuable insights into the potential benefits of such interventions. Luken and Sammons (2016) further supported these findings, emphasizing the positive association between mindfulness practices and reduced burnout among teachers. However, the existing literature predominantly focuses on general education settings, leaving a critical gap in our understanding of how mindfulness operates within the unique context of Chinese English as a Foreign Language (EFL) educators.

Abenavoli et al. (2013) underscored mindfulness as a protective factor against burnout, emphasizing its role in preserving teachers’ mental health. Despite these crucial insights, the literature lacks a nuanced exploration of cultural and contextual factors that may influence the efficacy of mindfulness interventions, particularly within the distinct landscape of Chinese EFL education. This study aims to address this gap by delving into the specific experiences of Chinese EFL educators, offering a more culturally sensitive understanding of the relationship between mindfulness, psychological capital, and teacher well-being.

Sun et al. (2019) explored the relationship between mindfulness and burnout among special education teachers, revealing mindfulness’s favorable influence on reducing burnout mediated by self-acceptance and perceived stress. While this study provides valuable insights, it is essential to consider the unique challenges faced by Chinese EFL educators, such as language barriers and diverse student demographics, which may introduce distinct stressors. Therefore, this study seeks to expand the current knowledge base by examining how mindfulness operates within the specific cultural and professional context of Chinese EFL education.

Roeser et al. (2013) advocated for mindfulness interventions to enhance teacher well-being, demonstrating a substantial reduction in teacher stress and burnout through mindfulness training. However, a critical examination reveals a gap in understanding the potential interplay between mindfulness and psychological capital—a key element that equips individuals with the psychological resources necessary to navigate and manage stressors effectively. This study aims to fill this void by investigating the combined influence of mindfulness and psychological capital on the well-being of Chinese EFL educators, providing a more comprehensive understanding of the factors that contribute to teacher resilience and satisfaction.

Overall, while the existing literature acknowledges the significant role of mindfulness in enhancing teacher well-being, a careful analysis reveals gaps in understanding its nuanced operation within the context of Chinese EFL education. This study aims to contribute to the field by exploring the relationship between mindfulness, psychological capital, and teacher well-being, offering culturally sensitive insights and effective strategies to promote the resilience and satisfaction of Chinese EFL educators, thereby addressing specific challenges within this educational setting.

### The research purpose

The primary aim of this study is to investigate the intricate relationship between psychological capital, teacher mindfulness, and teacher burnout. Two hypotheses are posited to elucidate this relationship comprehensively.

#### Hypothesis 1 (H1)

This hypothesis posits a direct and negative correlation between psychological capital and teacher burnout. The theoretical underpinning of H1 originates from the idea that an elevated level of psychological capital provides educators with essential resources to adeptly navigate the inherent stressors of the teaching profession (Kaya and Altınkurt, 2018). Psychological capital, encompassing elements such as self-efficacy, optimism, and resilience, theoretically empowers teachers to manage job demands effectively, thereby mitigating burnout (Luthans et al., 2007). This proposition finds support in empirical evidence, highlighting a consistent association between positive psychological resources and reduced levels of burnout among educators (Cheung et al., 2011; Hansen et al., 2015; Demir, 2018; Ferradás et al., 2019; Zhang et al., 2019; Freire et al., 2020).

#### Hypothesis 2 (H2)

This hypothesis suggests that teacher mindfulness mediates the relationship between psychological capital and teacher burnout. It posits that mindfulness, characterized by present-moment non-judgmental awareness of thoughts and emotions, serves as a mediator through which psychological capital influences teacher well-being (Flook et al., 2013; Hwang et al., 2017). The rationale behind H2 is rooted in the idea that psychological capital may facilitate the development of mindfulness practices. Teachers with higher psychological capital may be more inclined to employ positive coping strategies and demonstrate resilience conducive to effective mindfulness (Roche et al., 2014; Bi and Ye, 2021). Simultaneously, mindfulness has exhibited a negative correlation with teacher burnout, contributing to stress management, emotional regulation, and fostering a healthier perspective on work (Abenavoli et al., 2013; Flook et al., 2013; Roeser et al., 2013; Hue and Lau, 2015; Luken and Sammons, 2016; Sun et al., 2019; Ma et al., 2021). This hypothesis aligns with the concept of “self-regulation,” suggesting that individuals with greater psychological capital may possess enhanced self-regulation abilities, leading to improved mindfulness practices and subsequently reducing burnout (Baumeister and Heatherton, 1996).

Together, H1 and H2 are grounded in established theoretical frameworks and supported by empirical literature. They propose that psychological capital negatively impacts teacher burnout and that teacher mindfulness mediates this relationship. These hypotheses collectively provide a robust foundation to comprehensively explore the interplay among psychological capital, mindfulness, and teacher well-being.

## Materials and methods

### Participants and procedures

The selection of participants for this research study aimed at ensuring a representative and diverse sample of primary and secondary school teachers within the context of Chinese EFL education. The sampling process involved a multi-stage approach to capture a comprehensive snapshot of educators from various backgrounds. Initially, we collaborated with educational authorities in Chengdu, Sichuan province, China, to obtain a list of potential participating institutions. The selection of schools took into consideration factors such as geographical distribution, socioeconomic diversity, and educational levels to ensure a well-rounded representation. From this pool of potential institutions, a random sampling technique was employed to select participating schools, further enhancing the inclusivity of our sample. Once schools were identified, we worked closely with school administrators to obtain consent and cooperation for the study.

The recruited participants, comprising 387 primary and secondary school teachers, reflected a diverse demographic profile. These teachers, with a mean age of 38.7 years (SD = 9.8), were selected from a diverse demographic range, spanning from 24 to 57 years old. The gender distribution within the participant pool consisted of 146 male teachers (37.7%) and 235 female teachers (60.7%), while a small fraction of 6 participants (1.6%) did not adequately complete the questionnaire.

Considering the distribution across age brackets, 268 teachers (69.2%) were below the age of 45, with 119 teachers (30.8%) falling into the 45 years or older category. Moreover, in terms of teaching experience, 167 teachers (43.2%) had amassed over 11 years of teaching, while 220 teachers (56.8%) reported less than 11 years of teaching experience.

The data collection method involved administering a comprehensive 30-min paper-and-pencil survey in designated rooms within the participating educational institutions. Teachers were provided with a conducive environment to complete the survey, ensuring the collection of accurate and comprehensive responses. The completed questionnaires were then collected on-site to uphold anonymity and confidentiality for all participants. This approach facilitated a thorough gathering of data from a diverse group of teachers, allowing for a robust analysis of the variables under investigation within this study.

### Measures

#### Psychological capital

The assessment of Psychological Capital (PsyCap) involved the utilization of the PCQ survey designed by Luthans et al. (2015). This questionnaire (see the Appendix) employs a Likert scale spanning from 1 (indicating strong disagreement) to 5 (reflecting strong agreement) to gauge various facets of employees’ PsyCap, encompassing *self-efficacy*, *hope*, *resilience*, and *optimism*. An illustrative item from the PCQ includes the statement “Right now, I see myself as being pretty successful at work.”

#### Teacher burnout

The Maslach Job Burnout Inventory-Especial School (MBI-ES), devised by Maslach (1976), was utilized to evaluate teacher burnout (refer to the Appendix). Specifically adapted and applied to a Chinese teacher sample (Wu et al., 2003), the MBI-ES was translated from English to Chinese and subsequently revised employing rigorous psychological measurement techniques. This scale, comprising three dimensions—Emotional Exhaustion (EE), Personal Achievement (PA), and Depersonalization (DP)—elicited responses on a 5-point Likert scale, with 1 indicating “never happened” and 5 representing “almost happened every day.” The inventory consisted of 22 items collectively assessing EE (9 items), PA (5 items), and DP (8 items). The reliability and validity of this instrument have been rigorously examined within the Chinese context (Wang and Zhang, 2017). A sample item of the scale is “I look forward to teaching in the future.”

#### Mindfulness scale

Respondents’ mindfulness was assessed using the Five Facet Mindfulness Questionnaire (FFMQ-15) developed by Baer et al. (2012). This self-report questionnaire (see the Appendix) comprised 15 items, where participants rated their experiences on a 5-point Likert-type scale ranging from 1 (“never or very rarely true”) to 5 (“very often or always true”). A sample item of the scale is “I have trouble thinking of the right words to express how I feel about things.” Composite scores for mindfulness were derived by computing the mean of participants’ responses to the questionnaire items.

#### Data analysis

The initial exploration of the data involved calculating descriptive statistics and correlations using SPSS 28.0. This process provided a thorough overview of the dataset’s characteristics, helping us understand the distribution of variables and their relationships. Subsequently, Confirmatory Factor Analysis (CFA) was performed using AMOS 26.0 to assess the construct validity and verify the adequacy of the proposed model (Hair et al., 1998). Through this analytical approach, we could examine the expected connections between latent constructs and their observable indicators. Moreover, it allowed us to evaluate how well the proposed model aligned with the actual data collected.

Furthermore, Structural Equation Modeling (SEM) was employed to investigate the hypothesized relationships among latent constructs. This analytical technique allowed for a more in-depth exploration of complex relationships between variables, considering both direct and indirect effects (Kline, 2011). Evaluation of model fit utilized established fit indices recommended by Hu and Bentler (1999). These fit indices encompassed several metrics, including the ratio of χ^2^ goodness of fit to the degree of freedom (df). A favorable fit was indicated by a χ^2^/df ratio below 3, with a corresponding *p*-value exceeding 0.05. Moreover, Goodness of Fit Index (GFI) and Comparative Fit Index (CFI) values of 0.90 or higher were indicative of a well-fitting model. Additionally, Root-Mean-Square Error of Approximation (RMSEA) values below 0.08 and Standardized Root-Mean-Square Residual (SRMR) values below 0.10 were considered acceptable indicators of fit.

## Results

### Data screening and preprocessing

Before conducting the primary model testing, we performed a thorough data screening process using SPSS 28. This process included examining missing data, assessing normality, and detecting outliers, following established guidelines (Hair et al., 1998; Tabachnick and Fidell, 2007). While various techniques exist to manage missing data, such as list-wise deletion and pair-wise deletion, these methods may not be suitable for studies with smaller sample sizes or a significant amount of missing data. In this study, we employed the Expectation–Maximization (EM) algorithm (Kline, 2011) to handle missing data, as it provides imputation by replacing missing values. Furthermore, both univariate and multivariate outliers were scrutinized using Z-Standardized scores for univariate outliers and Mahalanobis $D^{2}$ for multivariate outliers (Tabachnick and Fidell, 2007). Following identification, these outliers were removed from the dataset to minimize their potential influence on subsequent analyses.

In this study, we conducted an investigation to examine the means and standard deviations of three variables among male and female educators. The average scores and standard deviations of burnout for males and females were 4.00 ± 0.75 and 4.12 ± 0.68, respectively. Concerning teacher psychological capital, males demonstrated an average score of 3.45 ± 0.80, whereas females showed an average of 3.30 ± 0.85. For mindfulness, the means were 4.25 ± 0.92 for males and 4.10 ± 0.88 for females. Subsequent independent samples t-tests indicated no significant differences between males and females in burnout (*t* = −0.821, *p* = 0.387), teacher psychological capital (*t* = 1.054, *p* = 0.295), and mindfulness (*t* = −0.987, *p* = 0.331).

### Construct validity

Following the initial data screening process, CFA was conducted to evaluate the construct validity of the measurement models employed in the study. The aim was to assess the suitability of the models through the utilization of goodness-of-fit indices. Initially, the measurement models representing latent constructs were tested; however, some of these initial models did not demonstrate a satisfactory fit with the collected data. Consequently, adjustments were made to enhance their compatibility with the observed data patterns.

To enhance the precision of these models, targeted modifications were incorporated. Specifically, two items from the mindfulness scale and three items from the psychological capital scale were excluded due to their factor loadings falling below the recommended threshold of 0.40. Furthermore, two correlational paths between error terms of two latent constructs were introduced as part of the model refinements. These adjustments were instrumental in enabling the final measurement models to attain an acceptable fit with the dataset, showcasing improved alignment with the observed data patterns. Comprehensive details regarding the fit indices and the implemented adjustments to enhance model adequacy are presented in Table 1.

**Table 1:** CFA results.

Constructs	χ^2^	df	χ^2^/df	*P*	CFI	RMSEA	SRMR	α
PsyCap	195.320	105	1.860	<0.001	0.978	0.040	0.031	0.87
Mindfulness	120.150	150	1.848	<0.001	0.981	0.038	0.027	0.85
Burnout	175.600	90	1.951	<0.001	0.971	0.053	0.042	0.89

CFI, Comparative Fit Index; RMSEA, Root Mean Square Error of Approximation; SRMR, Standardized Root Mean Square Residual; α, Cronbach’s alpha coefficient.

The descriptive statistics and Pearson’s correlations among the key constructs—psychological capital, mindfulness, and burnout—are presented in Table 2. Correlation analyses revealed significant positive associations between psychological capital and mindfulness (*r* = 0.48, *p* < 0.01), indicating a moderate positive relationship between these constructs. Conversely, significant negative correlations were observed between psychological capital and burnout (*r* = −0.43, *p* < 0.01), as well as between mindfulness and burnout (*r* = −0.39, *p* < 0.01), denoting moderate negative relationships between these pairs of variables.

**Table 2:** Descriptive statistics and correlations.

Constructs	Psychological Capital	Mindfulness	Burnout
Psychological Capital	1	0.48**	−0.43**
Mindfulness	0.48**	1	−0.39**
Burnout	−0.43**	−0.39**	1
Mean	3.21	3.74	3.93
Standard Deviation (SD)	0.69	0.58	0.71
Skewedness	−0.12	−0.22	0.11
Kurtosis	−0.07	−0.11	−0.09

*p < 0.05, **p < 0.01.

Analyzing the descriptive statistics of the examined constructs provided specific insights. Psychological capital achieved a mean score of 3.21 (*SD* = 0.69), while mindfulness received a mean score of 3.74 (*SD* = 0.58). In contrast, burnout exhibited a mean score of 3.93 (*SD* = 0.71). Skewness values, approximately −0.12 for psychological capital, −0.22 for mindfulness, and 0.11 for burnout, indicate minor deviations from perfect symmetry in their distributions. Additionally, kurtosis values, approximately −0.07 for psychological capital, −0.11 for mindfulness, and − 0.09 for burnout, suggest distributions slightly flatter than a normal curve, yet within the expected range for a normal distribution.

In summary, the analysis of these values indicates relatively minor deviations from normality within the distributions of the variables. These deviations are unlikely to significantly impact the validity of statistical analyses assuming normality, especially in the context of larger sample sizes. Therefore, despite slight departures from perfect symmetry and flatter distributions, the data meet the assumptions required for statistical analyses, ensuring the reliability of the findings within this study.

Structural equation modeling fter applying SEM to explore the mediation effect of mindfulness in the relationship between psychological capital and teacher burnout, the suitability of the proposed structural model was evaluated using various fit indices for both male and female teachers.

**Figure 1:**
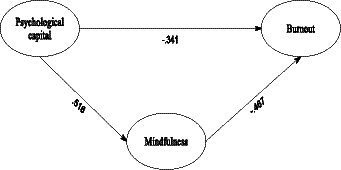
The structural model.

The results indicated that the proposed model exhibited favorable fit indices with the data for both male teachers (χ^2^/df = 1.522, CFI = 0.935, TLI = 0.931, IFI = 0.934, RMSEA = 0.042, SRMR = 0.054) and female teachers (χ^2^/df = 1.591, CFI = 0.932, TLI = 0.926, IFI = 0.932, RMSEA = 0.043, SRMR = 0.058). These indices suggested that the proposed model effectively represented the relationships between the variables, as illustrated in Figure 1, which displays standardized parameter estimates for the model.

Moreover, a multi-group invariance analysis was conducted to explore potential variations in model coefficients between male and female instructors. The χ^2^ difference test between constrained and unconstrained models (Δχ^2^ = 6.217, Δdf = 5, *p* = 0.344) indicated a similarity in the model coefficients across genders. Consequently, no significant discrepancies were observed between male and female instructors regarding both direct and indirect effects of the predictor variable on the outcome variable within the mediation model. Additionally, the utilization of bootstrap resampling with 500 iterations facilitated the assessment of the sampling distribution to further evaluate the indirect effects in both male and female groups.

The structural model proposed in this study, as presented in Table 3, demonstrates both the direct and indirect effects along with their corresponding 95% Confidence Intervals (CI). Examining the direct effects reveals significant pathways among the key constructs of psychological capital, mindfulness, and teacher burnout. Firstly, the analysis revealed a significant negative direct effect (*β* = −0.341, *p* < 0.001, 95% CI [−0.473, −0.209]) between psychological capital and burnout. This finding indicates that higher levels of psychological capital were associated with lower levels of burnout among teachers, suggesting a protective role of psychological resources against burnout.

**Table 3:** Direct and indirect effects of SEM analysis.

Model Pathways	Effect	Standard Error (*SE*)	Beta (*β*)	*p*-value	95% CI Lower Bound	95% CI Upper Bound
Psychological capital → burnout	Direct	0.142	−0.341	<0.001	−0.473	−0.209
Psychological capital → mindfulness	Direct	0.157	0.516	<0.001	0.282	0.750
Mindfulness → burnout	Direct	0.153	−0.467	<0.001	−0.613	−0.321
Psychological capital → mindfulness → burnout	Indirect	0.099	−0.240	<0.001	−0.320	−0.160

Beta (β), Standardized Coefficient; SE, Standard Error; CI, Confidence Interval.

Additionally, the pathway from psychological capital to mindfulness displayed a significant positive direct effect (*β* = 0.516, *p* < 0.001, 95% CI [0.282, 0.750]). This result indicates that greater psychological capital was linked to increased levels of mindfulness in educators, implying that higher psychological resources are conducive to fostering mindfulness practices among teachers. Similarly, the analysis revealed a significant negative direct effect (*β* = −0.467, *p* < 0.001, 95% CI [−0.613, −0.321]) between mindfulness and burnout. This finding indicates that higher levels of mindfulness were associated with reduced levels of teacher burnout, emphasizing the potential of mindfulness practices in mitigating burnout symptoms.

Furthermore, the investigation into the indirect effect of psychological capital on burnout through mindfulness mediation revealed statistical significance (*β* = −0.240, *p* < 0.001, 95% CI [−0.320, −0.160]). This significant indirect effect suggests that mindfulness partially mediates the relationship between psychological capital and teacher burnout. The identification of this indirect pathway underscores the mediating role of mindfulness in influencing the impact of psychological capital on mitigating burnout among educators.

To explore the consistency of path coefficients within the mediation model across distinct participant groups, a structural invariance test was executed, focusing on potential variations between male teachers and female teachers based on gender categorization. Analysis outcomes suggested that the proposed model demonstrated a commendable fit for both groups. Conducting a multi-group invariance analysis revealed that both the constrained (χ^2^/df = 1.620, CFI = 0.932, TLI = 0.927, RMSEA = 0.039, SRMR = 0.071) and unconstrained models (χ^2^/df = 1.618, CFI = 0.931, TLI = 0.926, RMSEA = 0.039, SRMR = 0.072) adequately represented the data patterns observed in male teachers and female teachers (see Table 4).

**Table 4:** Multi-group structural invariance analysis results.

Group	Model Type	χ^2^/df	CFI	TLI	RMSEA	SRMR
Male teachers	Constrained	1.620	0.932	0.927	0.039	0.071
	Unconstrained	1.618	0.931	0.926	0.039	0.072
Female teachers	Constrained	1.620	0.932	0.927	0.039	0.071
	Unconstrained	1.618	0.931	0.926	0.039	0.072

χ^2^/df, Chi-square to degrees of freedom ratio; CFI, Comparative Fit Index; TLI, Tucker-Lewis Index; RMSEA, Root Mean Square Error of Approximation; SRMR, Standardized Root Mean Square Residual.

Furthermore, the χ^2^ difference test (Δχ^2^ = 2.815, Δdf = 3, *p* = 0.480) indicated no substantial differences in model coefficients within the proposed mediation model between male teachers and female teachers based on gender categorization. This finding suggests a consistent pattern of relationships between variables across both gender groups.

Subsequent individual SEM analyses were performed for male teachers and female teachers (see Table 5), with both analyses demonstrating acceptable model fits for male teachers (χ^2^/df = 1.430, *p* < 0.001, CFI = 0.934, RMSEA = 0.050, SRMR = 0.069) and female teachers (χ^2^/df = 1.515, *p* < 0.001, CFI = 0.928, RMSEA = 0.053, SRMR = 0.067).

**Table 5:** Individual SEM analysis results.

Group	χ^2^/df	*p*-value	CFI	RMSEA	SRMR
Male teachers	1.430	< 0.001	0.934	0.050	0.069
Female teachers	1.515	< 0.001	0.928	0.053	0.067

These results collectively indicate the absence of significant differences in the direct and indirect effects of the predictor variable on the outcome variable between male teachers and female teachers based on gender classification. Moreover, the mediating role of the proposed mediator remained robust and consistent for both male teachers and female teachers, reinforcing the stability of the mediation model across gender categories.

## Discussion

The primary objective of this research was to investigate and delineate the intricate associations among psychological capital, mindfulness, and teacher burnout within educational contexts. This study sought to offer comprehensive insights into the roles played by psychological resources and mindfulness practices in influencing teacher well-being and mitigating burnout. By examining the relationships between these constructs, this research aimed to contribute to the existing body of knowledge surrounding the factors influencing teacher resilience and emotional health in educational settings. The study set out to explore the direct impact of psychological capital on teacher burnout while also examining the mediating role of mindfulness in this relationship.

The discovery of a direct and negative correlation between psychological capital and teacher burnout resonates strongly with established theoretical frameworks and empirical evidence in the field. This finding substantiates the theoretical foundations that propose higher levels of psychological capital as instrumental in providing individuals with essential psychological resources to adeptly navigate and manage stressors, effectively alleviating burnout (Luthans et al., 2007; Demir, 2018; Zhang et al., 2019). In alignment with previous research, this observed negative relationship between psychological capital and teacher burnout is consistently supported. Psychological capital, encompassing key elements such as self-efficacy, optimism, hope, and resilience, has consistently demonstrated a robust association with lower levels of burnout among educators across numerous studies (Cheung et al., 2011; Hansen et al., 2015; Demir, 2018; Kaya and Altınkurt, 2018; Ferradás et al., 2019; Zhang et al., 2019; Freire et al., 2020).

Grounded in positive psychology theory, the conceptual framework of psychological capital posits that individuals endowed with higher levels of these positive psychological resources are well-equipped to confront challenges, adapt to stressors, and maintain an optimistic perspective (Luthans et al., 2007; Newman et al., 2014; Luthans and Youssef-Morgan, 2017). This reservoir of positive attributes, as illuminated by the current study, serves as a protective mechanism potentially shielding teachers from the deleterious effects of burnout when faced with the rigors and demands inherent in their profession (Luthans et al., 2010; Kaya and Altınkurt, 2018; Zhang et al., 2019). The study’s findings, therefore, contribute to the robust evidence base supporting the crucial role of psychological capital in fortifying educators against burnout and fostering a resilient teaching workforce.

Theoretical frameworks supporting the protective role of psychological capital underscore the importance of possessing heightened psychological resources. For example, educators with amplified self-efficacy are more likely to set ambitious goals, show increased perseverance in the face of challenges, and demonstrate enhanced resilience to stressors. This collective fortitude acts as a buffer against burnout, allowing teachers to navigate the complexities of their profession more effectively (Skaalvik and Skaalvik, 2007). Similarly, psychological capital’s association with an optimistic outlook, hopeful disposition, and resilient nature has been instrumental in nurturing adaptive strategies. These strategies effectively mitigate stress and exhaustion, significantly reducing the likelihood of burnout among educators (Cheung et al., 2011; Kaya and Altınkurt, 2018; Ferradás et al., 2019; Freire et al., 2020).

In practical terms, teachers with higher psychological capital are better equipped to cope with the demands of their profession. They are more likely to persist in the face of setbacks, maintain a positive outlook, and proactively adapt to challenges. This not only safeguards their well-being but also enhances their capacity to provide quality education to students.

In addition, the direct and negative relationship between mindfulness and teacher burnout aligns with well-established theoretical frameworks. Mindfulness, as conceptualized by Kabat-Zinn (1994), involves present-moment awareness, non-judgmental observation of thoughts, and emotional acceptance. This fosters a more open and receptive approach to experiences. In the context of teaching, a profession known for its emotional demands and stressors, cultivating mindfulness becomes a potent resource.

Mindfulness practices enable educators to navigate challenges with increased resilience and emotional regulation. The ability to stay present in the moment, coupled with non-judgmental acceptance of thoughts and emotions, equips teachers with valuable tools to manage stress and emotional exhaustion effectively. This aligns with the findings that show mindfulness’s efficacy in promoting emotional regulation and reducing stress, ultimately mitigating burnout symptoms among educators (Roeser et al., 2013; Luken and Sammons, 2016; Sun et al., 2019).

Aligned with previous research, mindfulness practices demonstrate efficacy in promoting emotional regulation and reducing stress, thereby effectively alleviating burnout symptoms among educators (Flook et al., 2013 Roeser et al., 2013; Hue and Lau, 2015; Ma et al., 2021). Theoretical frameworks centered on mindfulness consistently advocate its role as a protective mechanism against burnout, enhancing teachers’ emotional management capabilities and fostering a more balanced psychological state (Abenavoli et al., 2013; Roeser et al., 2013; Hwang et al., 2017). Educators proficient in mindfulness exhibit an enhanced capacity to confront professional demands, adeptly navigate challenging situations, and maintain a healthier perspective, thereby reducing vulnerability to burnout (Roeser et al., 2013; Sun et al., 2019). Furthermore, the emphasis of mindfulness on present-moment attention and non-reactivity to thoughts and emotions appears to instill a sense of detachment from stressors among teachers. This detachment facilitates more adaptive responses to difficulties without succumbing to the emotional toll often associated with burnout (Abenavoli et al., 2013; Emerson et al., 2017).

The theoretical underpinnings of the observed relationship between mindfulness and teacher burnout are deeply rooted in the principles of mindfulness itself, as well as in established psychological theories. Mindfulness practices, as conceptualized by prominent figures like Kabat-Zinn (1994), emphasize cultivating present-moment awareness, non-judgmental observation of thoughts, and acceptance of emotions. These practices foster a mindset that enables individuals to engage with their experiences in a more open and receptive manner (Brown and Ryan, 2003; Kabat-Zinn, 2018, 2019). In the context of the teaching profession, characterized by high emotional demands and stressors, the cultivation of mindfulness emerges as a potent resource for educators. By developing present-moment awareness, teachers can better understand their own thoughts, emotions, and reactions in real-time. This heightened awareness allows them to respond to challenges with greater clarity and discernment, rather than reacting impulsively or being overwhelmed by stressors (Roeser et al., 2013; Luken and Sammons, 2016; Sun et al., 2019).

Furthermore, mindfulness practices facilitate emotional regulation, enabling teachers to manage their emotions more effectively. Through mindfulness, educators learn to observe their emotional experiences without immediately reacting to them. This non-reactive stance fosters a sense of emotional balance and resilience, reducing the likelihood of being swept away by negative emotions or becoming emotionally exhausted (Hülsheger et al., 2013). The integration of mindfulness into the daily lives of teachers reinforces the established belief that mindfulness equips them with invaluable tools to navigate the emotional landscape of their profession. By cultivating present-moment awareness, promoting non-reactivity, and enhancing emotional regulation, mindfulness practices empower educators to effectively manage the demands and stressors inherent in teaching. As a result, teachers are better equipped to prevent and alleviate burnout, thereby safeguarding their well-being and enhancing their professional resilience.

Finally, it was revealed that teacher mindfulness acted as a mediator between psychological capital and teacher burnout. This finding resonates profoundly with established theoretical frameworks and empirical evidence, illuminating the pivotal role mindfulness plays in mitigating the adverse effects of burnout by mediating the influence of psychological capital (Roche et al., 2014; Bi and Ye, 2021). Mindfulness, characterized by present-moment awareness, non-judgmental observation of thoughts and emotions, and a compassionate stance toward experiences, emerged as a crucial link between psychological capital and burnout (Kabat-Zinn, 1994; Roeser et al., 2013). This aligns with theoretical assertions that higher psychological capital endows individuals with the resources necessary to engage effectively in mindfulness practices, consequently reducing burnout tendencies (Roche et al., 2014; Bi and Ye, 2021).

Theoretical perspectives posit that psychological capital, comprising self-efficacy, optimism, hope, and resilience, enhances an individual’s capacity to cultivate and sustain mindfulness (Luthans et al., 2007; Luthans and Youssef-Morgan, 2017). Individuals endowed with higher psychological capital tend to demonstrate stronger self-regulatory abilities, increased resilience to stressors, and a positive outlook, collectively fostering the development of mindfulness (Luthans et al., 2010; Zhang et al., 2019). Mindfulness practices, in turn, empower teachers to effectively manage their emotional responses to stressors, acting as a buffer against the development of burnout (Roeser et al., 2013; Hwang et al., 2017). This mediation process suggests that teachers with elevated psychological capital are more inclined to engage in mindfulness practices, resulting in enhanced emotional regulation, reduced stress levels, and consequently lower burnout rates (Roeser et al., 2013; Hwang et al., 2017). Empirical studies consistently highlight the role of mindfulness in mitigating burnout by promoting emotional regulation and reducing stress among educators (Abenavoli et al., 2013; Flook et al., 2013; Roeser et al., 2013). The mediating role of mindfulness in the relationship between psychological capital and teacher burnout offers a comprehensive understanding of how these constructs dynamically interact and influence each other within the context of teacher well-being (Roche et al., 2014; Bi and Ye, 2021).

## Conclusion

This study has shed light on the intricate interconnections among psychological capital, mindfulness, and teacher burnout, offering valuable insights into the elements shaping teacher well-being within educational environments. The outcomes highlight the pivotal roles played by psychological capital and mindfulness strategies in alleviating burnout among educators. The established direct and negative correlation between psychological capital and teacher burnout is consistent with well-established theoretical frameworks and empirical evidence in this domain. Psychological capital, encompassing elements like self-efficacy, hope, optimism, and resilience, emerges as a shield against burnout. Educators equipped with higher psychological resources demonstrate enhanced capabilities to navigate the rigors and pressures inherent in their profession, thus reducing their susceptibility to burnout.

Moreover, this study delves into the mediating influence of teacher mindfulness on the association between psychological capital and burnout. Mindfulness practices, involving heightened awareness of the present moment and non-judgmental acceptance of thoughts and emotions, act as a conduit through which psychological capital impacts teacher well-being. Proficient mindfulness among teachers correlates with improved emotional regulation, effective stress management, and a more balanced perspective on their profession, consequently mitigating burnout tendencies.

The findings derived from this research possess substantial implications for educational practice, policy development, and future research trajectories. First and foremost, integrating professional development programs aimed at enriching teachers’ psychological capital and fostering mindfulness practices emerges as a pivotal avenue for educational institutions and policymakers. By equipping teachers with strategies to nurture their psychological resources and seamlessly integrate mindfulness into their routines, these programs can significantly contribute to fortifying teacher well-being and resilience. Establishing well-being initiatives within schools and educational organizations holds paramount importance. These initiatives should prioritize the mental health of teaching staff by offering mindfulness training, stress management programs, and interventions designed to augment self-efficacy and hope. Such initiatives can notably enhance the work environment’s health and bolster teachers’ coping mechanisms.

Furthermore, integrating mindfulness practices into the curriculum not only benefits teachers but also models mindfulness behaviors for students. By fostering a positive and stress-resilient school environment, curriculum-based mindfulness initiatives can foster a culture of well-being and emotional regulation among both teachers and students. Regarding policy development, recognizing the pivotal role of teacher well-being becomes imperative. Educational policies should address burnout rates by managing workloads, providing support for educators facing burnout, and introducing incentives to enhance psychological capital among teachers. Moreover, this study suggests potential directions for further research. While this research provides foundational insights, future studies employing longitudinal or experimental designs can establish causal relationships between psychological capital, mindfulness, and teacher burnout. Long-term investigations could refine intervention strategies and offer deeper insights into the effectiveness of specific interventions across diverse educational contexts.

The focus of this study on specific psychological factors is valuable, and there is an opportunity for future research to broaden the scope by exploring potential connections between the Chinese curriculum, workload, and teacher burnout. The demanding nature of standardized exams, extensive content coverage, and potential student pressure within the curriculum are factors that may significantly contribute to teacher stress and burnout. Including these aspects in future research endeavors would lead to a more comprehensive and holistic understanding of teacher well-being in the Chinese educational context.

In terms of educational policies, addressing burnout rates is crucial. This can be achieved by implementing strategies to manage workloads, providing robust support systems for educators facing burnout, and introducing incentives to enhance psychological capital among teachers. Furthermore, considering the potential influence of curriculum demands on teacher stress, future research could explore how curriculum design, workload management, and assessment practices can be optimized. This optimization should aim to prioritize teacher well-being while still achieving the educational goals set by the curriculum.

In acknowledging the valuable contributions of this study, it is imperative to critically assess its limitations, providing guidance for future research endeavors. The cross-sectional design employed in this study, while illuminating existing relationships, poses a constraint on establishing causality among the observed variables. To address this limitation, it is imperative for subsequent research to undertake longitudinal or experimental approaches, allowing for a more in-depth exploration of the causal pathways among psychological capital, mindfulness, and teacher burnout over time. Furthermore, the reliance on self-report measures in this study introduces the potential for response biases, as participants may provide socially desirable answers. Future investigations should consider incorporating a diverse range of data sources, including objective measures, to enhance the robustness and reliability of the findings. Employing a multi-method approach could offer a more comprehensive understanding of the complex relationships under scrutiny.

Considering the context specificity of the study, it is crucial for future research to broaden its scope by exploring varied educational settings. Investigating how these constructs manifest in diverse contexts will contribute to a more holistic understanding of the intricate interplay among psychological capital, mindfulness, and teacher burnout, thereby enhancing the generalizability of findings to different educational environments. Moreover, this study primarily focused on mindfulness as a mediator, potentially overlooking other mediating variables in the relationship between psychological capital and teacher burnout. Future studies should explore additional mediators to obtain a more nuanced and comprehensive understanding of the underlying mechanisms at play.

Additionally, the study did not delve into the specifics of mindfulness interventions, which presents an avenue for future research. Investigating the effectiveness of different components within mindfulness practices and variations in mindfulness programs can provide insights into tailored interventions for reducing teacher burnout. This research could inform the development of targeted and evidence-based strategies to enhance teacher well-being.

Addressing these limitations and building upon the findings will contribute to a more nuanced understanding of the intricate interplay between psychological capital, mindfulness, and teacher burnout. This, in turn, will facilitate the development of more effective strategies to promote teacher well-being and ultimately enhance the quality of education.

## Data availability statement

The data analyzed in this study is subject to the following licenses/restrictions: the raw data supporting the conclusions of this article will be made available by the authors, without undue reservation. Requests to access these datasets should be directed to RD, stevedu0530@sina.com.

## Ethics statement

The studies involving humans were approved by Jilin Normal University, Siping, China. The studies were conducted in accordance with the local legislation and institutional requirements. The participants provided their written informed consent to participate in this study.

## Author contributions

DL: Conceptualization, Investigation, Methodology, Project administration, Resources, Supervision, Validation, Writing – review & editing. RD: Conceptualization, Data curation, Formal analysis, Investigation, Methodology, Project administration, Resources, Software, Supervision, Validation, Visualization, Writing – original draft, Writing – review & editing.
