# Supplementary material for: Psychological capital, mindfulness, and teacher burnout: insights from Chinese EFL educators through structural equation modeling
Source: Front Psychol. 2024 Mar 14;15:1351912. doi: 10.3389/fpsyg.2024.1351912 (PMC10973147; doi:10.3389/fpsyg.2024.1351912)
Supplement: Supplementary file 1 [file Data_Sheet_1.docx]

**Supplementary File**

**Appendix**

***Psychological Capital Scale***

1 In this job, things never work out the way I want them to.

2 At this time, I am meeting the goals that I have set for myself.

3 I feel confident presenting information to a group of colleagues.

4 I feel confident helping to set targets/goals in my work area.

5 I can get through difficult times at work because I've experienced difficulty before.

6 There are lots of ways around any problem

7 I usually take stressful things at work in stride.

8 When I have a setback at work, I have trouble recovering from it and moving on.

9 When things are uncertain for me at work I usually expect the best.

10 I can be "on my own" so to speak at work if I have to.

11 If something can go wrong for me work-wise it will.

12 If I should find myself in a jam, I could think of ways to get out of it.

13 I feel I can handle many things at a time at this job.

14 I always look on the bright side of things regarding my job.

15 I feel confident contributing to discussions about the company's strategy.

16 I feel confident analyzing a long-term problem to find a solution.

17 Right now, I see myself as being pretty successful at work.

18 I’m always optimistic about my future.

19 I approach this job as if "every cloud has a silver lining”.

20 At the present time, I am energetically pursuing my work goals.

21 I feel confident contributing to discussions about the company's strategy.

22 I usually manage difficulties one way or another at work.

23 I feel confident contacting people outside the company (e.g., suppliers, customers) to

discuss problems.

24 I can think of many ways to reach my current work goals.

***Teacher Burnout Scale***

1. I look forward to teaching in the future.
2. I feel depressed because of my teaching experiences.
3. I get adequate praise from my supervisors for a job well done.
4. The teaching day seems to drag on and on.
5. I am glad that I selected teaching as a career.
6. The students act like a bunch of animals.
7. My physical illnesses may be related to my stress in this job.
8. I feel that the administrators are willing to help me with classroom problems, should they arise.
9. I find it difficult to calm down after a day of teaching.
10. Teaching is more fulfilling than I had expected.
11. I believe that my efforts in the classroom are unappreciated by the administrators.
12. If I had to do it all over again, I would not become a schoolteacher.
13. I feel that I could do a much better job of teaching if only the problems confronting me were not so great.
14. The stresses in this job are more than I can bear.
15. My supervisors give me more criticism than praise.
16. Most of my students are decent people.
17. Most students come to school ready to learn.
18. I feel that the administrators will not help me with classroom difficulties.
19. I look forward to each teaching day.
20. The administration blames me for classroom problems.
21. Students come to school with bad attitudes.

***Mindfulness Scale***

1. When I take a shower or a bath, I stay alert to the sensations of water on my body.
2. I’m good at finding words to describe my feelings.
3. I don’t pay attention to what I’m doing because I’m daydreaming, worrying, or otherwise distracted.
4. I believe some of my thoughts are abnormal or bad and I shouldn’t think that way.
5. When I have distressing thoughts or images, I “step back” and am aware of the thought or image without getting taken over by it.
6. I notice how foods and drinks affect my thoughts, bodily sensations, and emotions.
7. I have trouble thinking of the right words to express how I feel about things.
8. I do jobs or tasks automatically without being aware of what I’m doing.
9. I think some of my emotions are bad or inappropriate and I shouldn’t feel them
10. When I have distressing thoughts or images I am able just to notice them without reacting.
11. I pay attention to sensations, such as the wind in my hair or sun on my face.
12. Even when I’m feeling terribly upset I can find a way to put it into words.
13. I find myself doing things without paying attention.
14. I tell myself I shouldn’t be feeling the way I’m feeling.
15. When I have distressing thoughts or images I just notice them and let them go.
